# Development and Evaluation of a DAS-ELISA for Rapid Detection of Tembusu Virus Using Monoclonal Antibodies against the Envelope Protein

**DOI:** 10.1371/journal.pone.0096366

**Published:** 2014-05-05

**Authors:** Hao Chen, Quanbin Ou, Yi Tang, Xuhui Gao, Lili Wu, Cong Xue, Chunmei Yu, Jingteng Cui, Youxiang Diao

**Affiliations:** Poultry Diseases Lab, College of Animal Science and Technology, Shandong Agricultural University, Tai'an, Shandong, China; University of Texas Medical Branch, United States of America

## Abstract

Since April 2010, Tembusu virus (TMUV) which is a contagious pathogen of waterfowls, causing symptoms of high fever, loss of appetite and fall in egg production, has been reported in east of China. A double antibody sandwich enzyme-linked immunosorbent assay (DAS-ELISA) which detects for TMUV was developed, using two monoclonal antibodies (mAbs) against the TMUV envelope (E) protein. BALB/c mice were immunized with purified recombinant E protein expressed in *E. coli*. Three hybridoma cell lines designated as 12B1, 10C6 and 2D2, were screened by cell fusion and indirect ELISA for their ability to recognize different linear epitopes on the E protein, and were characterized subsequently. High-affinity mAbs 12B1 and 2D2 were used as capture and detection antibodies, respectively. The reaction conditions for the DAS-ELISA were optimized for TMUV detection. The cross-reactivity of the DAS-ELISA was determined using TMUV, duck plague virus, avian influenza virus subtype H9, Newcastle disease virus, duck hepatitis A virus type 1 and duck reovirus samples. A total of 191 homogenized tissues of field samples were simultaneously detected by DAS-ELISA and by RT-PCR. The former was found to have a high specificity of 99.1% and a sensitivity of 93.1%. These results reveal a positive coincidence between DAS-ELISA and RT-PCR at a coincidence rate of 95.8%. The method developed in this study can be used for the diagnosis of TMUV infection of duck origin.

## Introduction

A new infectious disease has been causing a sudden fall in the egg production of ducks from Jiangsu, Fujian, Anhui, Shandong, Hebei and other provinces in China since April 2010 [1]–[3]. The main pathological changes observed consistently in the affected ducks are ovarian hyperemia, hemorrhage, degeneration, distortion, macrophage and lymphocyte infiltration, and hyperplasia. Morbidity by this virus is usually high (up to 90%)[3], and mortality varies from 5% to 10%. The pathogen was identified by the NS5 gene sequence as TMUV, which belongs to the *Ntaya* virus (NTAV) group in the genus *Flavivirus* of the family *Flaviviridae* [1]. This duck TMUV disease has since caused an enormous loss in many layers and meat-type duck farms in China [2]–[5].

The mature virion of TMUV is 45 nm to 50 nm in diameter, coated with a capsule membrane, and contains a 10, 990 nt single-stranded positive-sense genomic RNA. The translation product of the genome is a polyprotein that contains three structural proteins (capsid, prM and envelope) and eight nonstructural proteins (NS1, NS2A, NS2B, NS3, NS4A, 2K, NS4B and NS5) [5]–[8]. The ‘pr’ peptide is cleaved from the prM structure during the maturation of flaviviruses, and the M protein remains in the mature particle as a transmembrane protein beneath the envelope (E) protein shell [9]. The ‘pr’ peptide was proposed to function as a cap-like structure that protected the fusion peptide on E from undergoing pre-mature fusion before virus release [10]. The E protein is the major surface protein of flaviviruses and is the dominant antigen that induces immunological responses in infected hosts and consequently elicits virus-neutralizing antibodies [11]–[13].

Several serological and molecular biological methods have been established, including reverse-transcription loop-mediated isothermal amplification (RT-LAMP) [14], [15], real-time RT-polymerase chain reaction (PCR) [16], blocking enzyme-linked immunosorbent assay (ELISA) [17]. These methods are not applicable both in directly antigen detection and large-scale molecular epidemiology under field conditions. TMUV has been detected in culex mosquitoes in China, in Thailand and Malaysia, indicating that mosquitoes may be important for transmission and that TMUV may have a large distribution outside of China. A new and effective detecting method is therefore urgently needed. This study aimed to develop a simple, convenient, time-saving, and cost-effective ELISA for the detection of TMUV. Monoclonal antibodies (mAbs) 12B1, 10C6, and 2D2 obtained in our study were able to recognize different linear epitopes on the E protein. A double-antibody sandwich enzyme-linked immunosorbent assay (DAS-ELISA) was established using high-affinity mAbs 12B1 and 2D2 as capture and detection antibodies, respectively. The assay demonstrated high sensitivity and specificity and may serve as an alternative method for detecting duck TUMV with its easy manipulation and low cost.

## Materials and Methods

### Ethics statement

The animal procedures were approved by the Animal Care and Use Committee of Shandong Agricultural University (Permit number: 20010510) and performed in accordance with the “Guidelines for Experimental Animals” of the Ministry of Science and Technology (Beijing, China). All surgery was performed according to recommendations proposed by the European Commission (1997), and all efforts were made to minimize suffering. Mice were housed in a temperature-controlled room with proper darkness-light cycles, fed with a regular diet, and maintained under the care of the Experimental Animal Center, College of Animal Science and Veterinary Medicine, Shandong Agricultural University. The mice were euthanized using sodium pentobarbital and the house sparrows were euthanized in CO_2_.

Duck and duckling samples were collected from Gaotang, Linyi, Pingyi and Feicheng counties, respectively. House sparrows were collected in Gaotang County and bred for further research. No specific permissions were required for these locations/activities. There are not endangered or protected species involved in these locations. GPS and companies of these lacations are listed as follows:

1. Gaotang County (36°50′14″N, 116°12′38″E), Company: Shandong Yonghui Food Limited Company, E-mail: liuhedhy@163.com.

2. Linyi County (37°10′33″N, 116°49′16″E), Company: Linyi Liuhe Jiusheng Limited Company, E-mail: dzlinyi-lc@liuhe.com.

3. Pingyi County (35°28′20″N, 117°44′25″E), Company: Pingyi Liuhe Food Limited Company, E-mail: 94723321@qq.com.

4. Feicheng County (36°4′37″N, 116°46′16″E), Company: Shandong Yonghui Food Limited Company, E-mail: hekangyuanhr@126.com.

### Relevant materials

Tembusu virus (TMUV), duck plague virus (DPV), H9 subtype avian influenza virus (AIV), Newcastle disease virus (NDV), duck hepatitis A virus-type 1 (DHAV-I) and duck reovirus (DRV) strains were collected in Shandong province and stored in Poultry Diseases Institute, Shandong Agricultural University. Purified recombinant E protein (40.5 KD) expressed in *E. coli* was obtained from Gao[18] and SP2/0 myeloma cell line was stored in our laboratory. BALB/c mice were purchased from the Experimental Animal Center of Shandong (Jinan, China).

### Production of mAbs to TMUV

Five 6-week-old female BALB/c mice were prepared as immunized animals. The mice were initially inoculated subcutaneously with 100 µg of immunogen (purified E protein) emulsified in an equal volume of complete Freund's adjuvant (Sigma, Missouri, USA). Immunogen emulsified in incomplete Freund's adjuvant (Sigma, Missouri, USA) was subsequently inoculated on the mice three times at 2-week intervals. Antiserum was collected from the lateral tail veins of immunized mice one week after the fourth inoculation and tested by indirect-ELISA to monitor that if the production of antibody was adequate for cell fusion. A final inoculation of immunogen without adjuvant was administered intraperitoneally four days prior to fusion. The mice were euthanized using sodium pentobarbital and sensitized spleen cells were fused with mouse myeloma cell SP2/0 using PEG1500 (Roche, Mannheim, Germany). The hybridomas were selectively cultured for approximately two weeks, and the cell supernatants were screened by ELISA against E protein expressed in *Escherichia coli* (*E.coli*). Positive hybridomas cultures were cloned at least twice by a limiting dilution in a 96-well cell culture plate.

The mAbs were generated by inoculating the selected hybridoma cells into BALB/c mice treated with sterile paraffin oil. Indirect ELISA, using E protein expressed in *E.coli* as coating antigen, was performed to titrate mAbs in the culture supernatants and ascitic fluids. The isotypes of the mAbs were determined by ELISA, using a mouse monoclonal antibody isotyping reagents kit (Sigma, Missouri, USA). The mAbs were purified from the ascitic fluids by ammonium sulfate precipitation. The concentration was determined by spectrophotometry.

### Characterization of mAbs

The reactions between mAbs and E protein were identified by western blot analysis. The E protein was separated by sodium dodecyle-sulfate polyacrylamide gel electrophoresis (SDS-PAGE) and transferred electrophoretically onto a polyvinylidene difluoride membrane (Roche, Mannheim, Germany). The membrane was blocked by the blocking buffer (PBS containing 0.5% (v/v) Tween-20 (PBST) and 2.5% skim milk powder) overnight at 4°C. Then the membrane was incubated with the purified mAbs diluted 1∶1000 in the blocking buffer, and goat anti-mouse IgG (H+L) (BoAoSeng Company, Beijing, China) was used to detect the bound antibodies. A colorimetric reaction was observed using 3, 3′-diaminobenzidine enhanced liquid substrate system (TianGen Corporation, Beijing, China).

The cross-reactivity of mAbs was examined by indirect ELISA. The mAbs were reacted with TMUV, but no reaction with DPV, AIV subtype H9, NDV, DHAV-I, and DRV were observed.

### Preparation of field samples

A total of 171 field samples from dead ducks suspected of TMUV infection and 20 house sparrows living around the infected duck farms were collected in Shandong province during 2010–2012 (Table 1). These duck farms are located in Gaotang, Linyi, Pingyi and Feicheng counties, respectively. House sparrows were collected in Gaotang County and euthanized in CO_2_ in our laboratory for further research. These tissue samples were stored at −80°C. All samples were homogenized in 5 mL of PBS containing penicillin (5, 000 U/mL) and streptomycin (5 mg/mL). The suspensions were subjected to three freeze–thaw cycles and centrifuged at 3, 000 g for 10 min. The supernatant samples were incubated for 15 min at 37°C before testing.

**Table 1:** Field samples collected from different duck farms in Shandong, China.

Species	Type of samples	Age(days)	No. of samples
laying duck a	theca folliculi	>150	137
duckling a	brain	>10	34
house sparrow b	brain	unknown	20
Total			191

a Samples were collected from dead ducks with symptoms of reduction in egg production (laying ducks), loss of appetite and encephalitis (ducklings).

b These samples were collected from house sparrow euthanized with CO_2_ near the TMUV-infected duck farms.

### Selection of pairing antibodies

mAbs were labeled with horseradish peroxidase (HRP, Sigma, Missouri, USA) by the sodium periodate oxidation method described by Kanpp [19]. The titers of the labeled mAbs were determined by indirect ELISA. The 96-well microtiter plates (Maxisorp Nunc, Denmark) were coated with purified capture mAbs diluted in sodium carbonate buffer. TMUV was added as antigen and the plates were incubated for 30 min at 37°C. Then mAbs conjugated with horseradish peroxidase (detection antibody) were added. The unbound conjugates were washed off after incubation, and 3, 3', 5, 5'-Tetramethylbenzidine (TMB) substrate solution (TIANGEN, Beijing, China) was added to each well. Incubation was carried out for 30 min and the reaction was stopped by adding 3M H_2_SO_4_. Plates were read at 450 nm on an automated ELISA plate reader (Bio-Rad, USA). The best pairing antibodies were obtained according to the recorded result.

### Development of DAS-ELISA

The optimal concentrations of capture mAb (5, 2.5, 1.25 and 0.625 µg/mL), detection mAb (1∶500, 1∶1000, 1∶1500, 1∶2000, 1∶2500 and 1∶3000), dilution of positive control (purified TMUV) and negative control (SPF duck serum) (1∶5, 1∶10, 1∶20, 1∶40, 1∶80 and 1∶160) were screened by checkerboard titration. DAS-ELISA was performed by the following procedure. Briefly, 96-well microtiter plates were coated with capture mAb (100 µL/well) diluted in 0.01 M PBS (pH 7.2) and incubated at 4°C overnight. The plates were washed three times with PBST (PBS containing 0.05% Tween-20), then applied with blocking buffer (200 µL/well) (1% bovine serum albumin (BSA), 5% skim milk, 5% fetal bovine serum (FBS), and 1% gelatin) at 37°C for 1 h. After washing three times with PBST, clarified field sample suspensions diluted 1∶2 in blocking buffer (100 µL/well) were added and incubated at 37°C for 1 h. Afterwards, the plates were washed three times with PBST and 100 µL diluted detection mAb conjugated to HRP was added to each well. After incubation at 37°C for 1 h and washing with PBST three times, TMB substrate solution (100 µL/well) was added to each well, and the plates were incubated in the dark at 37°C for 15 min. 3 M H_2_SO_4_ of 50 µL volume was added to stop reaction and the OD values were read at 450 nm using an automated ELISA plate reader. The optimal condition was obtained by comparing the positive/negative ratio (P/N) of the samples.

### Positive/negative cut-off value

Twenty-four negative sera from SPF ducks were detected by the established DAS-ELISA under the determined optimal conditions. The cut-off value at OD450 to define a virus positive was calculated based on the formula: positive and negative cut-off value  =  negative sample mean +3 standard deviations (mean+3 SD).

### Cross-reaction test

The specificity of the DAS-ELISA was evaluated by testing antisera to other avian pathogens including DPV, AIV, NDV, DRV, DHAV-1 and TMUV. The specificity of the method was evaluated based on the results.

### Duplicability test

Each sample was detected in parallel three wells in a 96-well plate coated with one batch of 12B1 (capture mAb) by DAS-ELISA for intra-batch assay (n = 10). These samples were detected by DAS-ELISA in 96-well plates coated with different batches of 12B1 for inter-batch assay. All these tests were repeated three times. The intra- and inter-assay coefficients of variation (%CV) were calculated by the following formula: %CV  =  standard deviations (SD)/ mean OD_450_ of samples ×100%.

### RT-PCR Assay

The RT-PCR assay used the recently described primers [20], and amplified a fragment of approximately 211 nucleotides, which corresponded to nucleotides 9340 to 9551 in the TMUV genome. The cycling protocol comprised an initial of denaturation at 94°C for 30 s, annealing at 55°C for 30 s, and extension at 72°C for 60 s, followed by a final elongation step at 72°C for 5 min. The PCR products were analyzed on 2.0% (w/v) agarose gel and stained with ethidium bromide. All the PCR products were cloned into a pMD18-T vector (Takara, Dalian, China) and sequenced (Invitrogen, Beijing, China).

### Comparison of DAS-ELISA and RT-PCR

A total of 191 field samples obtained from different duck farms in Shandong province were detected simultaneously by DAS-ELISA and RT-PCR. The sensitivity, specificity and accuracy were calculated by the following formulas: sensitivity =  true positive / (true positive + false negative) ×100%, specificity =  true negative/(true negative + false positive) ×100%, accuracy  =  (true positive +true negative)/ (true positive+ false positive + true negative + false negative) ×100%.

## Results

### Preparation and characterization of mAbs

The purified recombinant E protein was used as the immunogen. Cell culture supernatants were screened by indirect-ELISA for the presence of antibodies against E protein. After cell fusion, cell culture, antibody detection and subcloning, three mAbs designated as 12B1, 10C6 and 2D2 were obtained, and each recognized different linear epitopes on the E protein. The three obtained hybridoma lines were injected intraperitoneally into BALB/c mice to produce ascitic fluids. Their titer in the culture supernatant and ascites was 1∶400, 1∶400, 1∶800 and 1∶256000, 1∶128000, and 1∶512000 for 12B1, 10C6 and 2D2, respectively. Additionally, the isotype of the mAbs was IgG1, IgG1, and IgG2a, respectively. All of the three mAbs types contained κ light chains. The concentration of the purified mAbs was 2.181 mg/mL, 2.409 mg/mL and 2.233 mg/mL for 12B1, 10C6 and 2D2, respectively (Table 2).

**Table 2:** Properties of monoclonal antibodies against TMUV E protein.

MAbs	Isotype	Ascites titer	IgG yield (mg·mL^−1^)
12B1	IgG_1_, κ chain	1∶256000^a^	2.181
10C6	IgG_1_, κ chain	1∶128000	2.409
2D2	IgG_2a_, κ chain	1∶512000	2.233

a MAb titer was the last dilution that yielded an absorption value above 0.3 at 30 min after adding the substrate at room temperature.

### Reactivity and specific identification of mAbs

**Figure 1:**
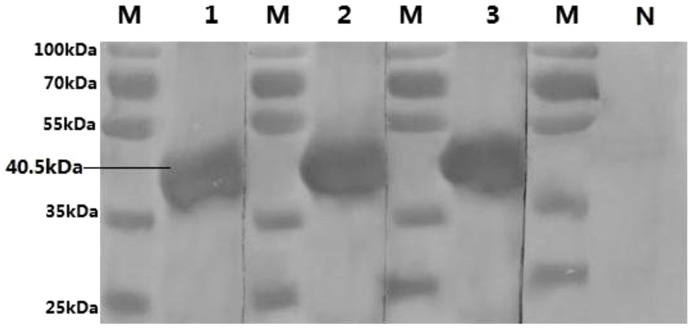
Reactivity of three mAbs with E protein by Western blotting. M, protein molecular markers; Lane 1, 2, 3 and N, purified recombinant TMUV E protein reacted with12B1, 10C6, 2D2 and SP2/0 cell supernatant, respectively.

Western blot was used to test the reactivity and specific identification of mAbs against the TMUV E protein. The corresponding analytic results demonstrated that the three mAbs reacted strongly with the TMUV E protein expressed in *E. coli* (Fig. 1) but did not react with other viruses that can also infect ducks (DPV, AIV, NDV, DRV and DHAV-I). Thus, the three mAbs were shown to be very specific for TMUV.

### Development of DAS-ELISA

mAb (12B1) and mAb (2D2) were determined as the optimal capture and detector antibodies by indirect ELISA, respectively. The 96-well plates were coated with 0.25 µg/well of purified mAb 12B1 (2.5 µg/mL) that was diluted in sodium carbonate buffer (pH 9.6). The plates were incubated at 4°C overnight. Dilutions of the positive and negative control were determined to be 1∶1000 and 1∶20, respectively. The most suitable blocking buffer concentration was 1% BSA. Using the 24 TMUV-antibody negative serum samples, the average OD value was determined to be 0.14±0.0153 (mean OD value± S.D.), resulting in a cut-off OD value of 0.2. No cross-reactions were detected by the DAS-ELISA using antisera against DPV, AIV, NDV, DRV and DHAV-1, with the OD values ranging from 0.099 to 0.124, demonstrating an excellent specificity of the DAS-ELISA for detection of the TMUV antigen (Table 3).

**Table 3:** Cross-reaction of DAS-ELISA.

Samples	DPV	AIV	NDV	DRV	DHAV-I	TMUV(+)	TMUV(−)
mean OD_450_ a	0.122	0.110	0.101	0.120	0.115	1.458	0.099

a Each sample was tested in two parallel repetitions by established DAS-ELISA. Samples except TMUV positive sera (OD450 values<0.2) were determined as negative.

### Stability of DAS-ELISA

The mean %CV of the intra- and inter-batch duplicability tests was 4.31% and 8.2%, respectively. The duplicability and stability of the developed DAS-ELISA were thus shown to be adequate for TMUV detection.

### Detection of TMUV in field samples

A total of 194 field samples of laying ducks, ducklings, house sparrows, and mosquitoes were screened for the presence of TMUV using DAS-ELISA and RT-PCR. In DAS-ELISA, 68 of the 194 field samples were determined to be positive, whereas 72 samples were positive by RT-PCR. Among the 194 samples, 67 were positive in both DAS-ELISA and RT-PCR and 118 were negative in both tests (Table 4). DAS-ELISA was found to be 99.1% specific (118/119) and 93.1% sensitive (67/72) relative to RT-PCR. The results of both DAS-ELISA and RT-PCR showed that the accuracy ((67+118)/191) of the two detection methods was 95.8%.

**Table 4:** Comparison of DAS-ELISA with RT-PCR for field samples detection.

DAS-ELISA	RT-PCR(+)	RT-PCR(−)	Total
Positive	67	1	68
Negative	5	118	123
Total	72	119	191

## Discussion

TMUV is an important pathogen in ducks in the south and east of China, and the early and rapid detection of TMUV is essential to prevent and control the spread of disease caused by the virus. In this study, three mAbs against TMUV E protein were obtained. A simple, convenient, and rapid DAS-ELISA procedure was established for the large-scale diagnosis of TMUV based on the three high- quality mAbs.

Monoclonal antibody is an important immunological tool. It has an important application in the determination of pathogen biological characteristics and antigenicity, as well as in pathogen detection, treatment, immune mechanism research, among others. Monoclonal antibody-based DAS-ELISA is a time-saving, conveniently operated method that has high sensitivity, specificity and efficiency. This method is also used extensively for the routine diagnosis of multiple pathogens, such as Influenza A virus [21], classical swine fever virus [22], and Peste Des Petits Ruminants Virus [23].

The E protein of the flavivirus is the largest structural protein and the main envelope glycoprotein that constitutes the virus surface protuberance. Under neutral and micro alkaline conditions, envelope protein is exciting in homologous dimer on mature virus [24]. The E protein that binds to cell receptors is the viral component that mediates the targeting of flaviviruses to vital host organs and has thus generated the highest interest in research [25]. The E protein also enables viral penetration during infection through the lipid endosomal membrane [26] and induces neutralizing, haemagglutination-inhibiting and protective antibodies [27]. The advantage of E protein based diagnostics is that the epitopes on the E protein are conserved among flaviviruses in previous studies. This feature helps avoid the overlooking of flavivirus infections from other geographical regions during detection.

In this study, BALB/c mice were immunized with purified TMUV emulsified with Freund's adjuvant. Three hybridoma cell lines were obtained and designated as 12B1, 10C6 and 2D2. These three mAbs are able to recognize three different linear epitopes on the E protein eliminating the competition of mAbs on the same epitope and minimizing steric hindrance. The DAS-ELISA assay was established by capture antibody 12B1 and detection antibody 2D2. The accuracy of the two detection methods was 95.8%. The developed method for TMUV detection was also shown to have no cross-reactivity with DPV, AIV, NDV, DRV and DHAV-I. These results collectively indicate that the developed DAS-ELISA method can be used for the survey and diagnosis of TMUV.

In summary, three mAbs against TMUV E protein were obtained in our study, and the established DAS-ELISA assay for TMUV was shown to be convenient and easy to perform. The DAS-ELISA assay is suitable for the diagnosis and antigen detection of clinical cases, investigation of duck TMUV.
